# The Rise of Clinical Decision Support Algorithms in Pain Management 2009–2024

**DOI:** 10.1007/s11606-025-09600-9

**Published:** 2025-05-12

**Authors:** Dan Kabella, Dorie Apollonio, Halle Young, Kelly R. Knight

**Affiliations:** 1 Center for Tobacco Control Research and Education, University of California San Francisco, San Francisco, CA USA; 2 School of Pharmacy, University of California San Francisco, San Francisco, CA USA; 3 Department of Humanities & Social Sciences, University of California San Francisco, San Francisco, CA USA; 4 School of Medicine, University of California, San Francisco, 490 Illinois Street, #71M, San Francisco, CA 94158 USA

**Keywords:** pain, opioid industry, clinical decision support, algorithms, opioid risk scores

## Abstract

This paper examines the rise of clinical decision support algorithms used to assess risk in pain management and the opioid industry’s influence on their development and implementation. To understand this influence, we conducted a qualitative study of documents related to the development of a tool that relied on artificial intelligence (AI) to suggest modifications in opioid prescribing, called NarxCare. The study began with keyword searches of the Opioid Industry Document Archive (OIDA), which contained over 3 million documents at the time of the study, to examine the pharmaceutical industry’s role in shaping the digital transformation of opioid prescribing. Our findings highlight industry-driven investments, educational campaigns, corporate policy activities, and the reliance on proprietary data that facilitated the widespread implementation of NarxCare. The increasing reliance on NarxCare raises concerns about its limited transparency, unknown reliability, and potential bias which may disproportionately affect certain patient groups based on race, socioeconomic status, or health conditions. This paper argues that the promotion of technologies like NarxCare allows the pharmaceutical industry to reinforce the narrative that opioids can be effective when prescribed responsibly, using advanced, data-driven strategies. Marketed as tools that assist clinicians in making more informed prescribing decisions, NarxCare contributes to the portrayal of the industry as a responsible actor in the regulation and distribution of opioids. Shifting attention to individual risk factors rather than systemic challenges enables the pharmaceutical industry to sidestep its role in the opioid crisis and evade scrutiny for its influence over regulation, the sponsorship of education and research, lobbying, supply chain control, and public health narratives. While NarxCare aims to improve prescribing safety, it requires critical evaluation in terms of effectiveness, ethical considerations, and the continued influence of the pharmaceutical industry in its design and implementation.

**Supplementary Information:**

The online version contains supplementary material available at 10.1007/s11606-025-09600-9.

## INTRODUCTION

Risk assessment tools have become integral to opioid prescribing strategies aimed at promoting patient safety. However, their influence extends beyond healthcare settings to pharmaceutical markets and regulatory frameworks. This raises concerns about the factors shaping the development and use of these tools, including the role of pharmaceutical companies, medical education, and the potential for bias in algorithmic decision-making. Historians have found that the pharmaceutical industry seeks to maintain a distinction between legally prescribed *medications* and illicit *drugs.*^1–4^ One way the pharmaceutical industry supports the continued use of opioids within regulated, legal medical frameworks—referred to as “white markets”—is by ensuring that opioids are prescribed under controlled conditions that include adhering to strict regulations and monitoring systems to minimize misuse while maintaining access for legitimate medical use.^1,5^ Promoting the legitimacy of opioids as medical treatments by regulating them is central to the development of data-driven electronic medical record-embedded tools. A popular tool intended to guide opioid prescribing decisions is NarxCare, which has been adopted in 52 of 54 US Prescription Drug Monitoring Programs (PDMPs).^6^ NarxCare employs an algorithm that aggregates and analyzes data from various sources, including PDMPs that are maintained by state governments. The algorithm is proprietary and claims to flag behaviors that may indicate risk of misuse, such as obtaining prescriptions from multiple doctors or pharmacies, commonly referred to as “doctor shopping.” The risk score and related alerts are intended to guide healthcare providers in making informed prescribing decisions. However, the proprietary nature of the algorithm means that the criteria and weighting of factors used to calculate risk are not publicly available. As a result, clinicians, legal scholars, and bioethicists have raised concerns about the tool’s lack of transparency and potential biases.^7–16^ The absence of rigorous, independent validation studies further complicates the evaluation of its clinical effectiveness and reliability.^11^ In this paper, we sought to describe and critically examine the expanding use of NarxCare to understand the role of clinical support algorithms in pain management and the opioid industry’s influence on its development and widespread adoption. We investigated how the tool may shift perceived responsibility for opioid misuse from opioid manufacturers and distributors to individuals.

## METHODS

For this content analysis, we examined industry documents from the Opioid Industry Document Archive.^17^ The UCSF-JHU Opioid Industry Documents Archive (OIDA) is a collaborative undertaking between the University of California, San Francisco and Johns Hopkins University that collects, organizes, preserves, and provides free online access to millions of previously internal documents made public through legal settlements to facilitate investigation into the opioid crisis. While not exhaustive, the archive provides valuable insights into the strategies and actions of opioid manufacturers, regulators, technology vendors, and other stakeholders that have influenced the rise of algorithms and risk assessment tools in prescribing practices. Since the 1990 s, industry documents have been critical to research that has identified and assessed commercial determinants of health.^18–20^

Between January and June 2024, we conducted keyword searches that began with terms such as “NarxScore.” The team, led by a trained science and technology studies scholar (DK), reviewed documents returned by the initial searches to identify additional search terms (a snowball search strategy) including the names of key actors (“James Huizenga,” “National Association of Boards of Pharmacy,” and “Appriss”), and “opioid safety” to examine the industry logic. Additional authors, with expertise in medical anthropology and a decade of NIH-funded research expertise on opioid prescribing in clinical settings (KK, HY), and public policy (DA) and two decades of experience conducting research using industry documents (DA), reviewed the findings and identified key themes in 1:1 and joint discussions until reaching consensus on which documents were relevant to the study objectives. In total, we identified 48 relevant documents, including emails and meeting agendas spanning 2009 to 2022. Of these, 20 documents provided key insights into decision-making processes and motivations that were not captured in media reports and are included in the findings below.

To triangulate our findings, we consulted company websites, patent texts, and news articles. Below we provide an overview of the opioid overdose crisis, the pharmaceutical industry’s role, and evolving regulatory responses. Our findings focus on three areas: (1) the rise of PDMP algorithms in opioid control, (2) industry-driven public health solutions, and (3) controversies surrounding the adoption of NarxCare.

## THE OPIOID OVERDOSE CRISIS: THE ROLE OF INDUSTRY AND EVOLVING REGULATORY RESPONSES

The US opioid overdose crisis is often framed in terms of four distinct “waves”: (1) prescription opioid overprescribing (2000–2009), (2) heroin-related overdoses (2010–2012), (3) the rise of synthetic opioids like fentanyl (2013–2014), and (4) the co-use of opioids and stimulants (2015–2021).^21,22^

The opioid crisis was shaped in part by the financial ties between the pharmaceutical industry and healthcare policymakers, which have influenced research, education, innovation, and clinical decisions that led to opioid overprescribing due to the profit motive of opioid manufacturers.^5,23–28^ Despite the introduction of federal drug control policies in the 2010 s, the pharmaceutical industry’s influence on prescribing has persisted. For example, in April 2011, the US Food and Drug Administration (FDA) introduced a class-wide Risk Evaluation and Mitigation Strategy (REMS) for long-acting and extended-release opioid analgesics. REMS sought to ensure the safe use of high-risk opioids by requiring *manufacturer*-led provider training, patient education, and risk management plans. REMS regulates opioids in the post-market phase, at the points of prescribing and dispensing. Manufacturers have been accused of designing weak REMs and downplaying issues that arise from risk-benefit assessments.^29^ The development and use of NarxCare partially reflected REMS’ efforts to balance opioid access with the need to reduce misuse and overdose by leveraging artificial intelligence to monitor prescription patterns and identify potential risks in real time. Opioid manufacturers’ involvement in designing REMS shaped regulations that were designed to ensure the safe use of opioids while allowing for their continued distribution. The adoption of algorithms into regulatory frameworks highlighted the growing influence of emerging technology on clinical practices.

## THE RISE OF PDMP ALGORITHMS IN OPIOID CONTROL, 2009–2014

In the USA, all 50 states (as well as 4 territories) operate independent, web-based PDMPs, with 48 requiring clinicians and/or pharmacists to check PDMPs before prescribing or dispensing medications.^30^ Not all states have the same PDMP requirements, and some have exceptions, such as not tracking certain prescriptions from out-of-state providers. In the 2000 s, PDMPs transitioned from paper to electronic format. This change was driven by policy, such as e-prescribing laws, and backed by trade associations (e.g., National Association of Chain Drug Stores) that sought to improve dispensing efficiency, reduce costs, and enhance patient safety.^31^

**Figure 1:**
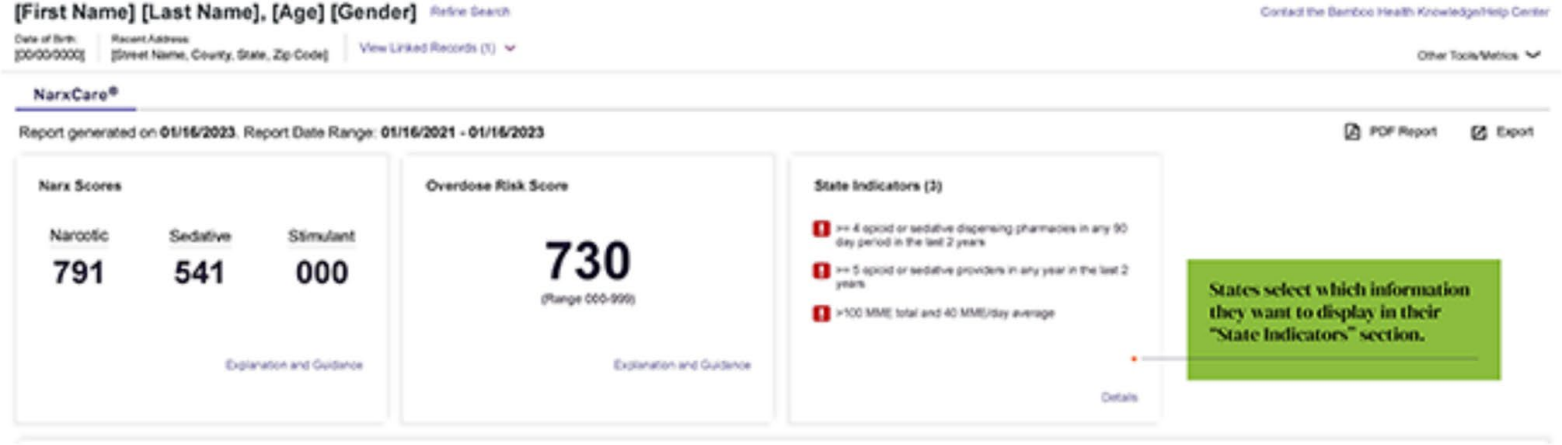
Optimize care coordination. A figure from Narxcare solutions’ promotional material illustrating the user platform interface. https://bamboohealth.com/solutions/narxcare/.

Private companies began marketing decision tools using the new electronic PDMPs to healthcare institutions as a way to improve prescribing through workflow enhancements and computerized alerts.^32,33^ A key policy milestone in this digital transformation was the 2009 Health Information Technology for Economic and Clinical Health (HITECH) Act, which allocated 25.9 billion USD to health information technology (health IT) infrastructure.^34^ Major industry players, including the pharmaceutical distribution company McKesson, actively lobbied for HITECH, anticipating benefits from the increased integration of technology in healthcare systems.^35^ The shift toward algorithm-driven decision-making accelerated in 2010 when an emergency physician and software developer in Ohio developed the first algorithm to assess the likelihood of “proper prescription drug use” by patients using PDMP data, which became NarxCare.^36,37^ PDMPs track controlled substance prescriptions at the state level and NarxCare is integrated with these systems to analyze and flag patterns of potential misuse (Fig. 1). The functionality of NarxCare is shaped by each state’s PDMP differences in data collection, regulations, participation, and data sharing, which impact its risk assessments and prescribing recommendations.

NarxCare was patented by Dr. James Huizenga, initially owned by the National Association of Board of Pharmacists, and then implemented locally by private vendor Eagle Software Corporation.^38^ The Substance Abuse and Mental Health Services Administration (SAMHSA) initiated a project in 2011 to improve access to PDMPs through health IT, in collaboration with the CDC and the Office of National Drug Control Policy. The 3-year project included six pilots with varying objectives in five states that tested the impact of enhanced PDMP access on clinical decision-making. For example, the Indianapolis study centered on integrating PDMP data into clinical workflows, while the Ohio study focused on the role of risk indicators in prescribing behavior.^39^

While integrated NarxCare Scores are designed to assist, rather than replace, clinical decision-making, prominent display and risk representation within electronic health records raise important concerns regarding user interface design.^13^ For instance, a study integrating a different machine learning algorithm into electronic health records highlighted the need for clear communication of risk to minimize biases and errors. It suggested that presenting risk in a more nuanced way—beyond persuasive elements such as simple categories (e.g., high risk) and color coding (e.g., red)—would help clinicians make more informed decisions.^40^ A senior pharmacy director testified in a 2021 opioid litigation regarding the use of NarxCare in pharmacies, expressing concern that pharmacists might make “knee-jerk” decisions based solely on the risk score, and potentially view it as the definitive factor in determining whether to fill a prescription.^41^ This testimony highlights the need for further research into the human-computer design of interactions within these systems, and education of NarxCare users about potential bias.

In June 2014, the National Association of Boards of Pharmacy, in partnership with Mallinckrodt Pharmaceuticals’ Anti-Diversion Industry Working Group, released a video titled *Red Flag*.^42^ This video, marketed as continuing education for pharmacists, exemplified how the pharmaceutical industry actively participates in shaping a narrative around opioid use. It frames the crisis as being driven by the actions of a few individuals—patients who misuse opioids, doctors who prescribe inappropriately, and drug dealers who traffic illicit opioids, while largely overlooking the industry’s role. This narrative downplays the influence of pharmaceutical companies that encouraged prescribers to treat pain as “the fifth vital sign” and made misleading claims to patients that suggested opioids were essential for maintaining quality of life after injury or as a normal part of aging.^43,44^

The corporate-sponsored educational video for pharmacists emphasized patient behaviors—such as visiting multiple prescribers or paying with cash—as signs of potential drug diversion. Coupled with the suggestive subtitle “How to Spot a Doctor Shopper,” it reflected the industry’s effort to reinforce a narrative that opioid misuse was primarily the result of individual patient actions, rather than addressing deceptive advertising and aggressive marketing by the industry, as well as the industry’s lobbying of physicians and regulators to broaden the definition of appropriate opioid use to include conditions like chronic pain. Pharmaceutical companies also failed to disclose the risks of addiction and overdose, while inadequate oversight enabled widespread overprescription and misuse.^45^

This framing may deflect responsibility from the pharmaceutical industry, positioning it as a steward of the legitimate opioid market and reinforcing its role in controlling access to prescription opioids.^46^ In this context, clinical algorithms such as NarxCare that rely on PDMP data, alongside educational tools like *Red Flag*, contribute to a broader narrative that shapes clinical understandings of opioid misuse, medical necessity, and the role of the pharmaceutical industry in managing opioid-related risks. In some cases, this could introduce criminal-legal frameworks into healthcare settings, inadvertently placing pharmacists in roles where they are expected to police patients.^47,48^

## INDUSTRY-SPONSORED ACTIVITIES AND TECH POLICY, 2016-2021

The growing role of commercial tools in shaping federal opioid policy coincided with increasing controversy over the industry’s role in the opioid overdose crisis. In response to public scrutiny, opioid manufacturers attempted to shift the blame for the crisis by reinforcing a narrative that misuse was primarily caused by patients and healthcare practitioners, while claiming that the risks of opioids could be mitigated through responsible prescribing. These efforts were complemented by their actions in funding initiatives aimed at enhancing PDMPs. The promotion of NarxCare by technology vendors, pharmaceutical companies, and policymakers facilitated its rapid integration into clinical use, despite limited supporting evidence. Educational initiatives also extended the influence of tools like NarxCare.

In March 2016, at the National Rx Drug Abuse and Heroin Summit in Atlanta, then-President Obama addressed healthcare professionals and people with opioid use disorder with a plan of action that made the incorporation of PDMPs central to addressing the opioid crisis.^49^ The summit highlighted strategies that relied on PDMP data, with NarxCare featured as a key tool. Bamboo Health (NarxCare’s vendor as of 2021) hosted a break-out session and promoted the tool as the “next-generation solution” for PDMPs, which would increase collaboration between the private and public sectors.^49^

A 2016 study sponsored by Bamboo Health and conducted by paid consultants found that NarxCare Scores were an “effective determinant of the risk of unintentional overdose death (see Appendix I).” However, the study’s validation focused solely on the prediction performance using a narrow sample of unintentional overdose deaths in Ohio.^50^ This narrow approach may have overlooked the broader range of factors influencing overdose deaths across different regions or demographic groups.

**Figure 2:**
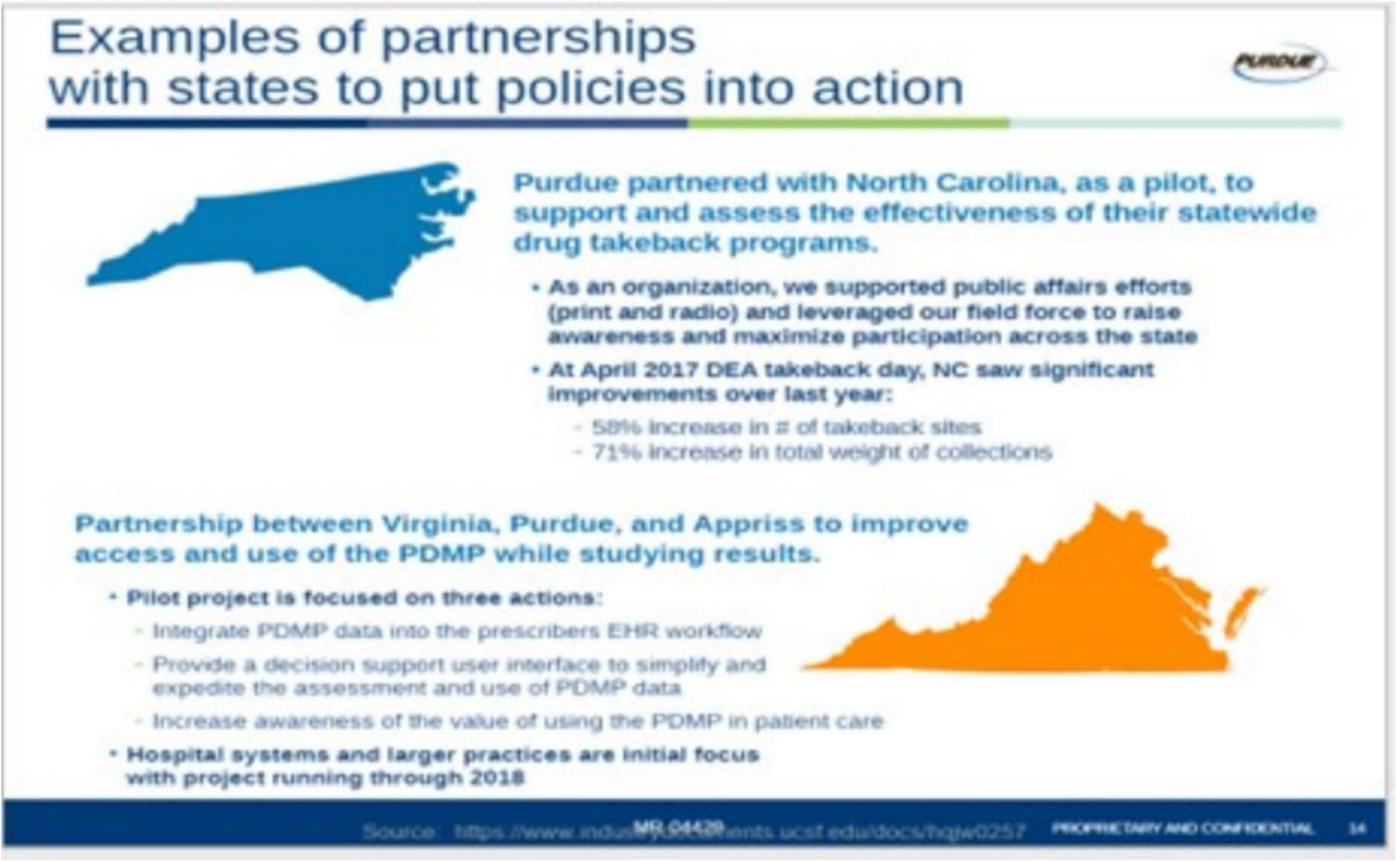
Examples of partnerships with states to put policies into actions. A slide from “Partnering with Purdue, Our Commercial Approach: Supporting Responsible Opioid Prescribing”. Source: McKinsey Documents, Industry Documents Library. https://www.industrydocuments.ucsf.edu/docs/hqjw0257.

In January 2017, former Virginia Governor McAuliffe announced a $3.1 million grant from Purdue Pharma to establish the “NarxCare bridge” between electronic health records systems and the PDMP.^51^ The project focused on three actions: (1) integrating PDMP data into prescriber workflow, (2) enhancing user interfaces to simplify data use, and (3) increasing awareness of PDMP’s value in patient care (see Fig. 2).^52^

Bamboo Health sponsored the 2017 National Rx Drug Abuse & Heroin Summit. The company led a workshop that promoted NarxCare as a way to address substance use disorders and improve patient outcomes.^53^ The summit brought together experts in law enforcement, policy-making, and frontline healthcare workers, offering continuing education credits to participants.

Similarly, in 2017, Purdue Pharma, advised by the consulting firm McKinsey & Company, introduced strategies in a PowerPoint presentation titled “Partnering with Purdue, Our Commercial Approach: Supporting Responsible Opioid Prescribing.” The presentation included a section labeled “Responsible Opioid Prescribing,” outlining the company’s approach, which included distributing tear pads, fact sheets, and posters that conveyed the risks of opioid misuse and offered guidance on how to address these issues using PDMP data.^52^ Although these commercial strategies may align with established clinical practices, manufacturer-led PDMP initiatives raise important questions about the perception and management of opioids within healthcare systems. These initiatives may introduce clinical norms that align with the goals outlined in manufacturer-developed factsheets and tear pads.

The growing influence of the opioid industry was further evidenced by the establishment of the National Patient Safety Network in 2017. This multistakeholder alliance, which included technology startups, medical associations, opioid industry affiliates, and patient groups worked to position NarxCare as a “complete solution” to address opioid overprescribing.^54^ The alliance developed a “standardized approach to PDMPs” recommending “targeted and efficient interventions” into prescribing practices.^55^ The plan, outlined in PowerPoint presentations, white papers, and meeting notes, is detailed in a series of emails exchanged between January and November 2017.^54–58^ The proposed plan aimed to complement state PDMPs by delivering real-time clinical alerts, using “NarxCheck” (the original branding of NarxCare) to providers and pharmacists to aid decision-making. It also proposed to facilitate the exchange of prescription drug monitoring data across state lines, leveraging existing technology, standards, and procedures.

**Figure 3:**
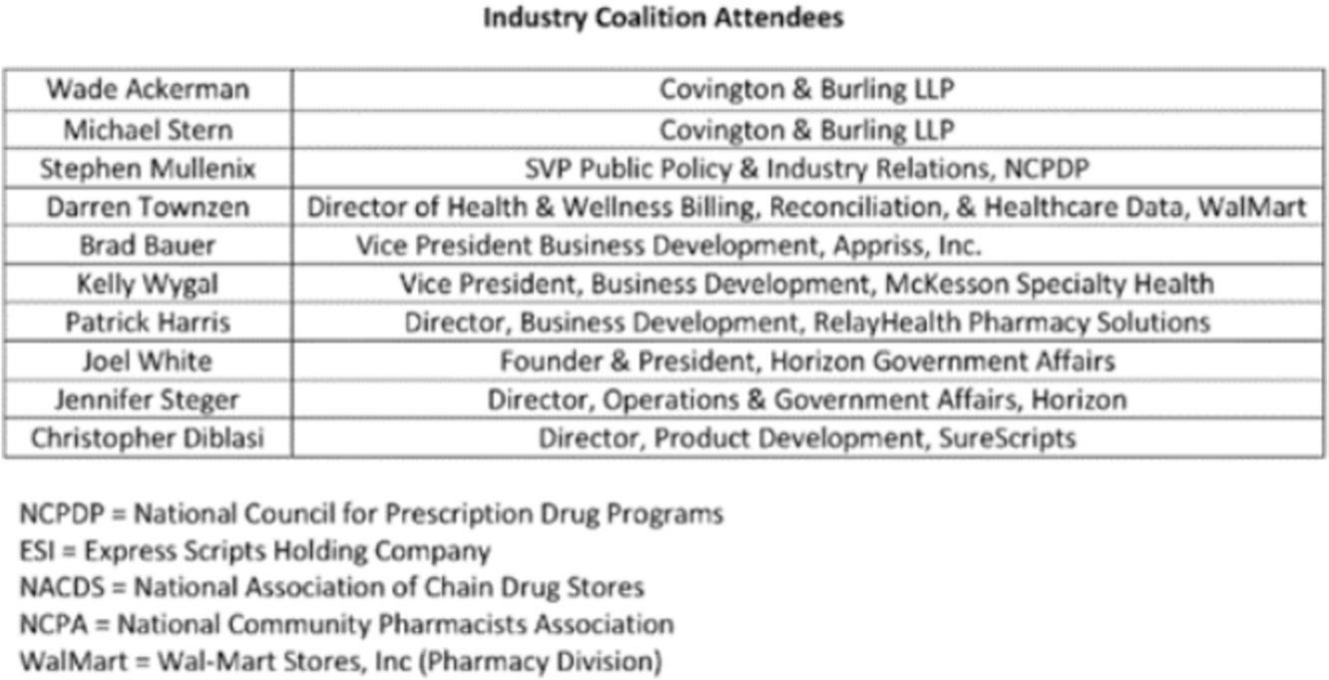
Industry Coalition attendees. An email attachment from coalition notes after the December 3, 2017 FDA Meeting in at the White Oak Campus in Silver Spring, MD. Source: Mallinckrodt Litigation Documents Collection, Industry Documents Library, https://www.industrydocuments.ucsf.edu/docs/gmmp0236.

To advance its goals, an industry special interest group within the alliance, known as the “Industry Coalition” (see Fig. 3 for list of members and affiliations), took a proactive step in March 2017.^54^ The group met with the FDA to advocate for the agency to use its existing REMS authority to mandate risk assessment using the proposed plan. Additionally, they identified and discussed other regulatory pathways.^57^ By framing their approach as distinct from law enforcement’s control over the informal market, the Industry Coalition sought to position NarxCare as a “complete solution” for prescribing practices. In meeting agenda notes, they emphasized that the “[Patient Safety Network] is clinical in nature, not a law enforcement effort.” To build broader consensus around this idea, they proposed engaging in both public forums and private meetings.^58^

In parallel to these efforts, In October 2017, Joel White, founder and president of Horizons Government Affairs and a member of the Industry Coalition, sent an email titled “Opioid Emergency,” urging members to take advantage of the increasing public scrutiny of the FDA’s opioid policies, writing:

While some believe now is the time to duck and cover, we hold the opposite view, the reporting will create a desire for Congress and the Administration to “get something done.” Positioning efforts to take advantage of this environment is important as we believe proactive solutions will save lives. At all times we recommend conveying a clear understanding of the severity of the opioid crisis and our shared commitment to identifying and advancing solutions.^59^

The top action item on the six-item “get something done“ list was to establish a “fully interoperable Prescription Drug Monitoring Program.” This was coupled with strategies to mobilize the Patient Safety Network solution at a congressional briefing titled “Using Health IT to Combat the Opioid Crisis” with an “aggressive media campaign” to sway Congress, the Trump Administration, and the public. White ended the email with a final call for action: “Now is not the time to be shy. The [*Washington Post*] and 60 Minutes reporting and the ongoing tragic crisis present opportunities to advance solutions that will save lives.”^59^

**Figure 4:**
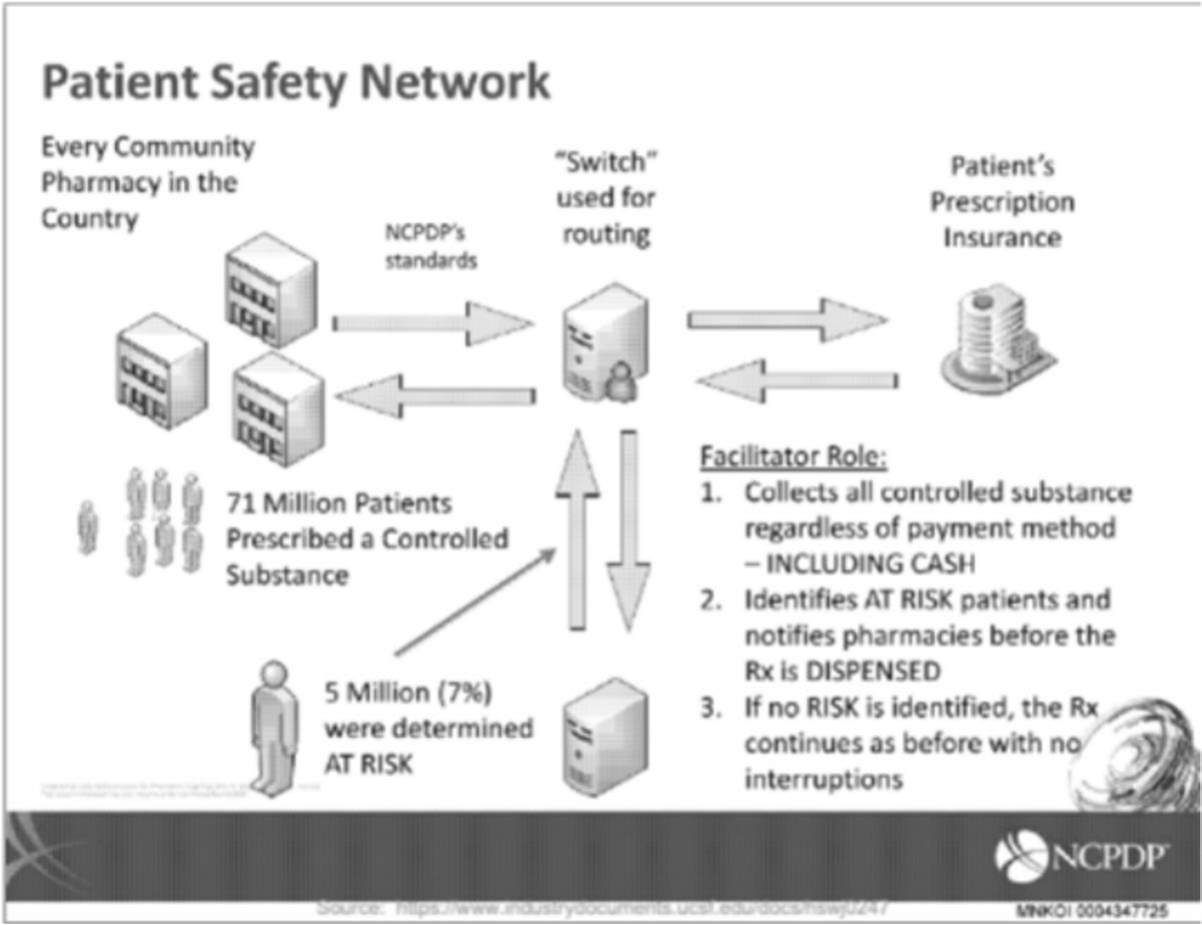
Patient Safety Network. A slide from “Industry re National Safety Network” a presentation given at the December 3, 2017 FDA Meeting in at the White Oak Campus in Silver Spring, MD. Source: Mallinckrodt Litigation Documents Collection, Industry Documents Library, https://www.industrydocuments.ucsf.edu/docs/hswj0247.

In 2018, this initiative culminated in the formal establishment of “Opioid Safety Alliance,” a rebranded iteration of the Patient Safety Network (see Fig. 4).^60^ A central focus of this alliance was to pass the “Analyzing and Leveraging Existing Rx Transactions (ALERT) Act” (still pending as of 2024), with McKesson committing to invest in developing a national prescription safety alert system as outlined in the Act^61^ According to their website, McKesson’s plan would integrate a real-time alert system into *pharmacist workflows* to address gaps in clinical decision support systems at point of *dispensing*. ^62^ A key provision in the ALERT Act directs the FDA to exercise its REMS authority to “*require* opioid manufacturers to provide controlled substances *only to* pharmacies and healthcare providers *that participate* in a prescription safety alert system.” While McKesson viewed the requirement for manufacturers to release opioids only to participating pharmacies and healthcare providers as a “positive step forward,” it also raises concerns about the coercive power inherent in corporate political activity.^63^ This form of structural power, where opioid access is tied to participation in safety alert systems, may pressure providers to conform to industry-driven protocols that favor manufacturer profits over patient-driven outcomes. Manufacturers may leverage this control to extend their influence beyond continuing education into healthcare access which reinforces their authority on legitimate opioid use.

## NarxCare CONTROVERSY: NAVIGATING THE TENSION BETWEEN CARE AND REGULATION, 2021–2024

Public controversy regarding NarxCare first appeared with anecdotal reports that drew significant attention to the potential harm of its algorithmic risk assessments. In 2021, *WIRED* magazine published a cover story that highlighted the experiences of women with complex conditions (e.g., endometriosis and chronic pain) who were reportedly denied pain medications based on high NarxCare scores.^64^ These anecdotal accounts underscored the human cost of the system’s influence, as patients with legitimate medical needs appeared to have been penalized by an algorithm that had not been scientifically validated.

The professional response to these concerns was more formal and structured. In 2022, the President of the California Society of Addiction Medicine and 21 colleagues sent a letter to the California Department of Justice (which administers the PDMP for the state) referencing the *WIRED* article. The letter raised concerns about NarxCare’s opaque, proprietary AI and machine learning algorithms, focused on their potential impact on prescribing practices, and cited the lack of FDA review and independent research.^65^ The letter highlighted how proprietary control limited transparency and created uncertainty in clinical decisions. These claims are central to research on commercial determinants of health that focus on corporate strategies. One corporate tactic conceptualized by historian of science Robert Proctor is the “production of ignorance,” a term that describes how industries deliberately obscure or suppress information and thereby produce ignorance or uncertainty of outcomes.^66^ Whether intentional or unintentional, such obscuring undermines both accountability and progress, allows potentially harmful products to circulate without scrutiny, and hinders opportunities for improvement.

By 2025, NarxCare was used in 52 of 54 US PDMPs.^67^ In response to growing concerns about NarxCare’s potential risks, the Center for US Policy took a more direct legal approach and submitted a citizen petition with the FDA in April 2023.^68^ The petition argued that NarxCare should be classified as a misbranded device and recalled from the market, citing its potential to restrict access to pain relief and drive patients toward unregulated black market opioids.^69,70^ The petition focused on the regulatory shortcomings of NarxCare and the broad range of data points the algorithm may consider in generating a risk profile. These data points include not only health records but also, in some cases, criminal records and financial information, raising questions about privacy, data security, and the potential for biased or unfair assessments. Technology can perpetuate biases when it draws on criminal justice systems that have been repeatedly demonstrated to operate with bias.^71–74^ By incorporating data from criminal justice sources, NarxCare risks reinforcing discriminatory practices and healthcare inequalities and potentially subjects individuals with prior criminal justice involvement to biased risk assessments in opioid prescribing.^7,75^

The different responses to NarxCare’s impact—anecdotal evidence, professional letters, and formal legal petitions—reveal the complexity of the controversy. Examining the NarxCare controversy within the broader scope of the pharmaceutical industry’s influence on opioid prescribing practices highlights a troubling dynamic. By prioritizing individual risk factors, NarxCare supports the industry’s narrative that the opioid crisis is primarily a matter of personal responsibility, deflecting attention from the industry’s systematic role in exacerbating the crisis. This shift in focus enables pharmaceutical companies to mitigate accountability while positioning opioids as acceptable when prescribed in accordance with a proprietary risk scoring system that may reinforce existing bias. The lack of independent scientific research on NarxCare’s clinical effectiveness underscores the need for further investigation and regulation regarding its use and impact on health outcomes.^7,11^

## DISCUSSION

Risk assessment tools like NarxCare play a key role in promoting safe opioid prescribing, yet their clinical impact remains unclear. This uncertainty extends beyond clinical practice, as these tools also influence access to opioids and highlight the role of the opioid industry in shaping healthcare provider decisions and regulation. The integration of tools like NarxCare into clinical decision-making, using data drawn from both PDMPs and other sources, has allowed pharmaceutical companies to influence the management and monitoring of prescribed opioids, which may help maintain their influence over the market for opioids.

PDMPs and associated algorithms such as NarxCare are designed to monitor and regulate opioid prescriptions within a “white market” that shapes perceptions of opioid use and safety based on individual patient behavior that is labeled “criminal” or “compliant.”^1,5^ In this way, these tools not only track prescriptions but are part of a larger industry context. Reliance on tools such as NarxCare contributes to a narrative surrounding opioid consumption, medical necessity, and safety that claims that opioid companies are trustworthy and dependable but patients are not.

This paper identifies several key themes regarding the emergence of tools like NarxCare in opioid markets. First, the opioid industry may influence, through corporate policy activities and legislation (such as the 2009 HITECH Act, the ALERT Act, and FDA’s REMS), the regulatory landscape that affects opioid prescribing and healthcare practices. These initiatives have facilitated the integration of tools like NarxCare into clinical workflows, potentially shaping regulatory guidelines and clinical decision-making around opioid prescribing. This has been framed around the idea that patients and healthcare practitioners were primarily responsible for opioid misuse, while pharmaceutical companies promoted the view that the risks of opioids could be managed through responsible prescribing.

Second, despite limited evidence of its effectiveness, industry-driven investments and educational campaigns have led to the widespread adoption of NarxCare. In promoting tools with unknown validity, vendors undercut independent data and critical perspectives on their tools’ effectiveness. The reliance on proprietary data and trade secrets about algorithmic design limits transparency, which hinders accountability and allows potentially harmful products to circulate without opportunities for improvement.

Third, scientific evidence highlights biases in machine learning algorithms and AI across clinical settings broadly, raising concerns about the accuracy, fairness, and reliability of outcomes. This is particularly relevant for populations that have faced historical discrimination based on socioeconomic status, health conditions, and race. Further research is essential to assess the implications for health equity of widespread NarxCare use in prescribing. Scientists have called for national policy discussions on opioid prescribing practices, particularly in resource-limited settings.^76^ Our study on clinical algorithms adds depth to these conversations by showing that the role of AI in shaping the evolution of these practices has been both influential and complex, highlighting the need for thoughtful integration that balances technological advancement with equitable healthcare delivery.

Our study has limitations. Although OIDA held over 3.1 million documents as of July 2024 and offers a unique and valuable perspective on opioid industry activities, it remains an incomplete record of industry activities. Some documents have been redacted due to trade secret protections, and companies not involved in litigation related to the opioid epidemic were not required to release records. These factors may limit the generalizability of our findings. Despite these constraints, OIDA provides critical information that is unavailable through other sources, presenting a rare opportunity to study industry influence on healthcare practices. OIDA plays a key role in critically evaluating tools like NarxCare and their links to industry influence, by offering access to a wide range of documents detailing the actions of opioid manufacturers, regulators, technology vendors and relevant stakeholders.

## CONCLUSIONS

This study examined the rise of clinical algorithms in opioid prescribing, with a focus on the design and deployment of NarxCare. The development and expansion of NarxCare over time highlights how corporate interests may influence the implementation of clinical algorithms to maintain an industry narrative that individual behavior, rather than industry marketing, led to the opioid crisis, and that controlling individuals is the appropriate strategy to address the crisis. The findings also raise broader lines of inquiry about the role of machine learning technologies in clinical care, suggesting that machine learning tools in healthcare should undergo the same rigorous evaluation relating to design and deployment as other health interventions. Concerns about NarxCare’s impact are valid and may be heightened by the incentives of the industries that contributed to its development. Given the limited evidence supporting the use of clinical algorithms in opioid prescribing, along with the potential for machine learning to introduce biases, our findings suggest that vendors that market tools like NarxCare should be transparent regarding how the tools generate recommendations. Furthermore, these tools should not be implemented widely before there is a clear understanding of their potential health impacts.

## Supplementary Information

Below is the link to the electronic supplementary material.

Supplementary file1 (DOCX 549 KB)

## Data Availability

The opioid industry documents used and analyzed during the current study are available to the public and can be found in the UCSF-JHU Opioid Industry Documents Archive, https://www.industrydocuments.ucsf.edu/opioids/. Unique persistent identifiers are provided for each cited document in the list of references.
