# Supplementary material for: The Rise of Clinical Decision Support Algorithms in Pain Management 2009–2024
Source: J Gen Intern Med. 2025 May 12;40(10):2423–32. doi: 10.1007/s11606-025-09600-9 (PMC12343441; doi:10.1007/s11606-025-09600-9)

**APPENDIX I**: NARxCHECK Score as a Predictor.pdf from Derek Naten’s “Top of Personal Folders Archive.” Source: <https://www.industrydocuments.ucsf.edu/docs/fxfj0247>
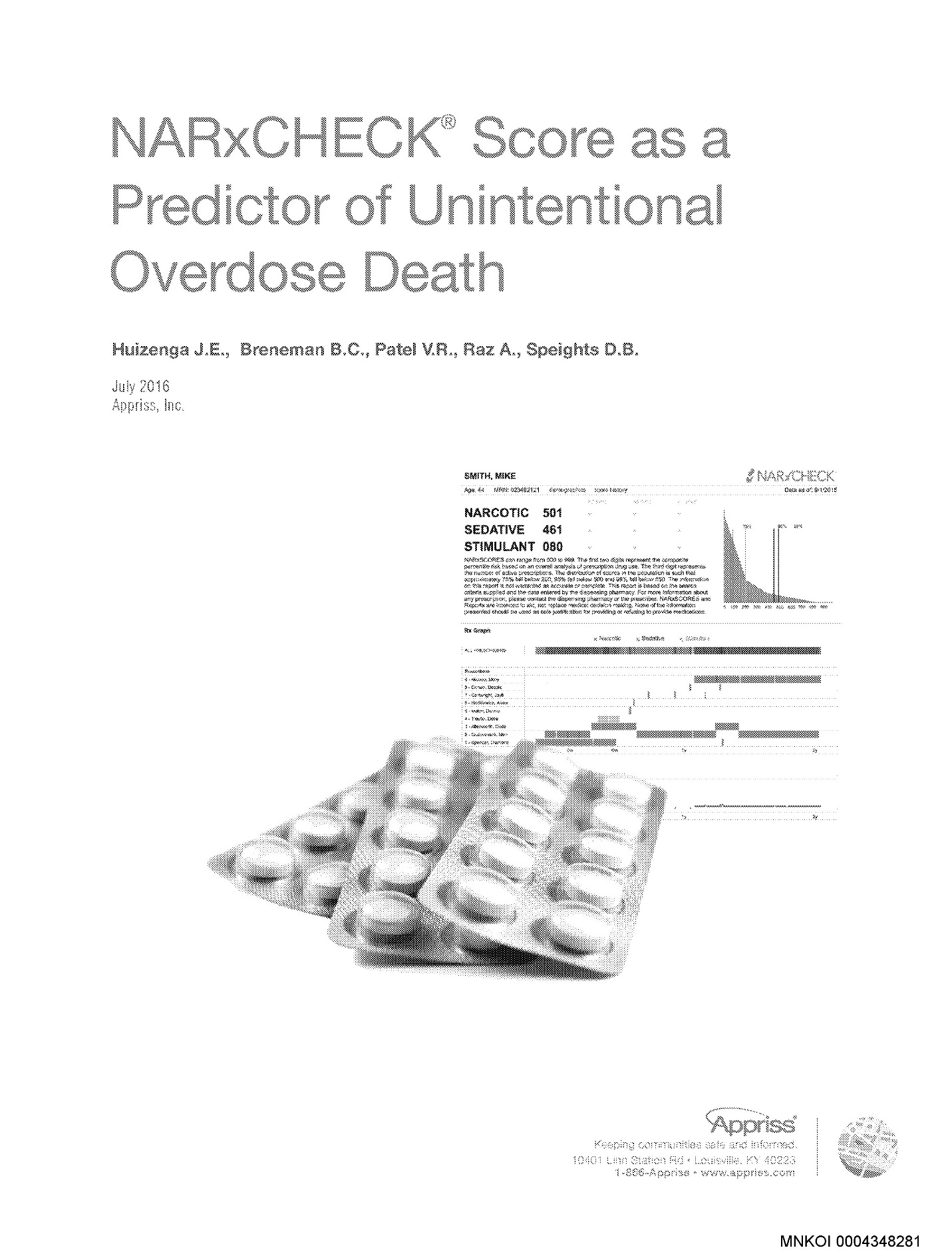


**APPENDIX II:**

**Supplementary Information** Key events in the historical emergence of clinical algorithms in opioid prescribing (Attachment)


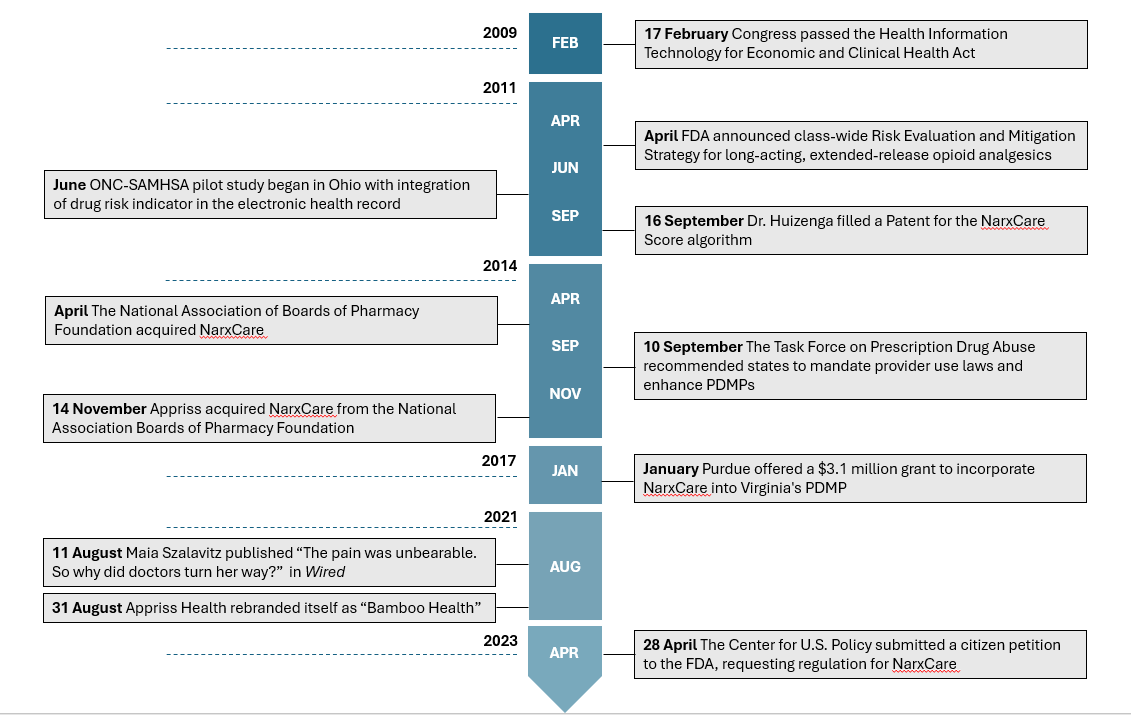

Supplement: Supplementary file 1 — Supplementary file1 (DOCX 549 KB) [file 11606_2025_9600_MOESM1_ESM.docx]
